# Supplementary material for: Control of carbon monoxide dehydrogenase orientation by site-specific immobilization enables direct electrical contact between enzyme cofactor and solid surface
Source: Commun Biol. 2022 Apr 26;5:390. doi: 10.1038/s42003-022-03335-7 (PMC9042819; doi:10.1038/s42003-022-03335-7)
Supplement: Supplementary file 2 — Supplementary Information [file 42003_2022_3335_MOESM2_ESM.pdf]

<u>**Supplementary information**</u>

Control of carbon monoxide dehydrogenase orientation by site-specific immobilization enables direct electrical contact between enzyme cofactor and solid surface

*Stacy Simai Reginald, Hyeryeong Lee, Nabilah Fazil, Basit Sharif, Mungyu Lee, Min Ji Kim, Haluk Beyenal, and In Seop Chang**

Table of Contents

Tables

Supplementary Table 1. Root mean square deviation value for the synthetic CODH-L against native CODH-L. 3

Supplementary Table 2. Root mean square roughness values of the bare gold substrate and the enzyme-modified gold substrates 4

Supplementary Table 3. List of primers used in this study 5

Supplementary Table 4. List of plasmids and strains used in this study 6

Supplementary Table 5. Amino acid sequence of native and synthetic CODH-L used in this study………......7

Figures

Supplementary Figure 1. Uncropped and unedited SDS-PAGE image of native and synthetic CODH-L....8

Supplementary Figure 2. Three-dimensional images of **a** bare gold surface and the cross-sectional profile along the line (lower panel), **b** native CODH-L, **c** CODH-L_{gbpN}, **d** CODH-L_{gbpN}, and **e** CODH-L_{gbpNC} when 0.5 µM of enzyme solution was incubated with gold substrate. 9

Supplementary Figure 3. Height histograms obtained from the AFM images for **a** native CODH-L, **b** CODH-L_{gbpN}, **c** CODH-L_{gbpC}, and **d** CODH-L_{gbpNC} when of 1 µM of enzyme solution was incubated with gold substrate. 10

Supplementary Figure 4. AFM images, 3D-surface profiles, and cross-sectional heights of **a** the bare screen-printed gold electrode surface (SPGE), **b** native CODH-L, **c** CODH-L_{gbpN}, **d** CODH-L_{gbpC}, and **e** CODH-L_{gbpNC} on the SPGE. 11

Supplementary Figure 5. a Representative CV profile of CODH-L_{gbpNC} showing the dependence of the DET current on the scan rates (scan rates: 20 to 100 mVs^{-1}). **b** Linear dependence of peak current and the square root of the scan rate. **c** CV profiles of the CODH-L_{gbpNC} when scanned continuously over 5 cycles in the presence of CO. **d** Amperometric response at an applied potential of −0.1 V (vs Ag/Ag^+) in the absence (blue diamond) and in the presence of CO (grey line). 12

Supplementary Figure 6. Variation of peak potential, E_p against the logarithm of scan rates, log *v*. …...…13

Supplementary Figure 7. CV profiles of **a** native CODH-L, **b** CODH-L_{gbpN}, **c** CODH-L_{gbpC}, and **d** CODH-L_{gbpNC} on the SPGE when methylene blue was added as the redox mediator in the absence (dot line) and presence of CO (solid line)... 14

Supplementary Tables

Supplementary Table 1. Root mean square deviation value for the synthetic CODH-L against native CODH-L.

Construct information	Root mean square deviation (RMSD) (Å)			
	PyMol	SuperPose	Chimera	Biophyton
CODH-L_{gbpN}	0.26	0.23	0.43	0.24
CODH-L_{gbpC}	0.21	0.23	0.26	0.24
CODH-L_{gbpNC}	0.25	0.27	0.26	0.29

Supplementary Table 2. Root mean square roughness values of the bare gold substrate and the enzyme-modified gold substrates

Construct type	Root mean square (RMS) roughness, R_q (nm)
Bare Au	1.32
CODH-L	2.58
CODH-L_{gbpN}	3.43
CODH-L_{gbpC}	3.33
CODH-L_{gbpNC}	8.31

Supplementary Table 3. List of primers used in this study

Primer name	Sequence	Information
ST053_F	AAAGCTAGCATGAACGCGCCGGTTCAGGATGC	Forward primer containing restriction site *NheI* to clone *cutL* into pET21a(+)
ST054_R	AAACTCGAGCAGCGCCAGGTTGTGTTCTTTCAG	Reverse primer containing restriction site *NheI* to clone *cutL* into pET21a(+)
ST150_F	TCTGCCGTCTGGCGGTGGTGGCGGCATGAACGCGCCGGTTCAG	Forward primer to add gbp and glycine linker at N-terminus
ST151_R	CGAGACGGCGGCAGGTGAGCTTTCAGGCTAGCCATATGTATATCTCCTTCTTAAAG	Reverse primer to add gbp and glycine linker at N-terminus
ST152_F	CCTGCCGCCGTCTCGTCTGCCGTCTCTCGAGCACCACCACCAC	Forward primer to add gbp and glycine linker at C-terminus
ST153_R	TGAGCTTTCAGGCCGCCACCACCGCCCAGCGCCAGGTTGTGTTC	Reverse primer to add gbp and glycine linker at C-terminus

[*] Underlined segments refer to the restriction enzymes used.

Supplementary Table 4. List of plasmids and strains used in this study

Strain/Plasmid	Relevant characteristics	Source
E. coli DH5α	F– φ80lacZΔ M15 Δ (*lacZYA-argF*) *U169 recA1 endA1 hsdR17* (rK– mK$^+$) *phoA supE44* λ- *thi*–1 *gyrA96 relA1*	Invitrogen
E. coli BL21 (DE3)	F– *ompT* *hsdS*$_B$ *(r*$_B$*–, m*$_B$*–) gal dcm (DE3)*	Invitrogen
E. coli BL21-AI	F$^-$*omp*T *hsd*S$_B$ (r$_B^-$ m$_B^-$) *gal dcm ara*B::T7RNAP-*tet*A	Invitrogen
pET21a (+)	Bacterial expression	Novagen
pET28a (+)	Bacterial expression	Novagen
pET21a::*cutL*	pET21a (+) carrying *cutL* gene	This work
pET21a::*gbp(N)_cutL_6XHis*	Expression plasmid carrying gbp *at* N-terminus and *cutL* gene	This work
pET21a::*cutL_gbp(C)_6XHis*	Expression plasmid carrying *cutL* gene and gbp at C-terminus	This work
pET21*a*::*gbp(N)_cutL_gbp(C)_6XHis*	Expression plasmid carrying *cutL* gene and gbp at C- and N-terminus	This work

Supplementary Table 5. Amino acid sequence of the native and synthetic CODH-L produced in this study.

Entry	Amino acid sequence
CODH-L_{wt}	MNAPVQDAEARELALAGMRPRACAKEDARFIQGKGNYVDDIKMPGMLHMDIVRAPIAHG<br>RIKKIHKDAALAMPGVHAVLTAEDLKPLKLHWMPTLAGDVAAVLADEKVHFQMQEVAIVI<br>ADDRYIAADAVEAVKVEYDELPVVIDPIDALKPDAPVLREDLAGKTSGAHGPREHHNHIFT<br>WGAGDKAATDAVFANAPVTVSQHMYYPRVHPCPLETCGCVASFDPIKGDLTTYITSQAPHV<br>VRTVVSMLSGIPESKVRIVSPDIGGGFGNKVGIYPGYVCAIVASIVLGRPVKWVEDRVENIST<br>TAFARDYHMDGELAATPDGKILGLRVNVVADHGAFDACADPTKFPAGLFHICSGSYDIPRA<br>HCSVKGVYTNKAPGGVAYRCSFRVTEAVYLIERMVDVLAQKLNMDKAEIRAKNFIRKEQFP<br>YTTQFGFEYDSGDYHTALKKVLDAVDYPAWRAEQAARRADPNSPTLMGIGLVTFTEVVGA<br>GPSKMCDILGVGMFDSCEIRIHPTGSAIARMGTITQGQGHQTTYAQIIATELGIPSEVIQVEEG<br>DTSTAPYGLGTYGSRSTPVAGAAIALAARKIHAKARKIAAHMLEVNENDLDWEVDRFKVK<br>GDDSKFKTMADIAWQAYHQPPAGLEPGLEAVHYYDPPNFTYPFGIYLCVVDIDRATGETKV<br>RRFYALDDCGTRINPMIIEGQIHGGLTEGYAVAMGQQMPFDAQGNLLGNTLMDYFLPTAVE<br>TPHWETDHTVTPSPHHPIGAKGVAESPHVGSIPTFTAAVVDAFAHVGVTHLDMPHTSYRVW<br>KSLKEHNLAL
CODH-L_{gbpN}	LKAHLPPSRLPSGGGGGMNAPVQDAEARELALAGMRPRACAKEDARFIQGKGNYVDDI<br>KMPGMLHMDIVRAPIAHGRIKKIHKDAALAMPGVHAVLTAEDLKPLKLHWMPTLAGDVAA<br>VLADEKVHFQMQEVAIVIADDRYIAADAVEAVKVEYDELPVVIDPIDALKPDAPVLREDLA<br>GKTSGAHGPREHHNHIFTWGAGDKAATDAVFANAPVTVSQHMYYPRVHPCPLETCGCVAS<br>FDPIKGDLTTYITSQAPHVVRTVVSMLSGIPESKVRIVSPDIGGGFGNKVGIYPGYVCAIVASI<br>VLGRPVKWVEDRVENISTTAFARDYHMDGELAATPDGKILGLRVNVVADHGAFDACADPT<br>KFPAGLFHICSGSYDIPRAHCSVKGVYTNKAPGGVAYRCSFRVTEAVYLIERMVDVLAQKLN<br>MDKAEIRAKNFIRKEQFPYTTQFGFEYDSGDYHTALKKVLDAVDYPAWRAEQAARRADPN<br>SPTLMGIGLVTFTEVVGAGPSKMCDILGVGMFDSCEIRIHPTGSAIARMGTITQGQGHQTTY<br>AQIIATELGIPSEVIQVEEGDTSTAPYGLGTYGSRSTPVAGAAIALAARKIHAKARKIAAHML<br>EVNENDLDWEVDRFKVKGDDSKFKTMADIAWQAYHQPPAGLEPGLEAVHYYDPPNFTYPF<br>GIYLCVVDIDRATGETKVRRFYALDDCGTRINPMIIEGQIHGGLTEGYAVAMGQQMPFDAQG<br>NLLGNTLMDYFLPTAVETPHWETDHTVTPSPHHPIGAKGVAESPHVGSIPTFTAAVVDAFAH<br>VGVTHLDMPHTSYRVWKSLKEHNLAL
CODH-L_{gbpC}	MNAPVQDAEARELALAGMRPRACAKEDARFIQGKGNYVDDIKMPGMLHMDIVRAPIAHG<br>RIKKIHKDAALAMPGVHAVLTAEDLKPLKLHWMPTLAGDVAAVLADEKVHFQMQEVAIVI<br>ADDRYIAADAVEAVKVEYDELPVVIDPIDALKPDAPVLREDLAGKTSGAHGPREHHNHIFT<br>WGAGDKAATDAVFANAPVTVSQHMYYPRVHPCPLETCGCVASFDPIKGDLTTYITSQAPHV<br>VRTVVSMLSGIPESKVRIVSPDIGGGFGNKVGIYPGYVCAIVASIVLGRPVKWVEDRVENIST<br>TAFARDYHMDGELAATPDGKILGLRVNVVADHGAFDACADPTKFPAGLFHICSGSYDIPRA<br>HCSVKGVYTNKAPGGVAYRCSFRVTEAVYLIERMVDVLAQKLNMDKAEIRAKNFIRKEQFP<br>YTTQFGFEYDSGDYHTALKKVLDAVDYPAWRAEQAARRADPNSPTLMGIGLVTFTEVVGA<br>GPSKMCDILGVGMFDSCEIRIHPTGSAIARMGTITQGQGHQTTYAQIIATELGIPSEVIQVEEG<br>DTSTAPYGLGTYGSRSTPVAGAAIALAARKIHAKARKIAAHMLEVNENDLDWEVDRFKVK<br>GDDSKFKTMADIAWQAYHQPPAGLEPGLEAVHYYDPPNFTYPFGIYLCVVDIDRATGETKV<br>RRFYALDDCGTRINPMIIEGQIHGGLTEGYAVAMGQQMPFDAQGNLLGNTLMDYFLPTAVE<br>TPHWETDHTVTPSPHHPIGAKGVAESPHVGSIPTFTAAVVDAFAHVGVTHLDMPHTSYRVW<br>KSLKEHNLALGGGGGLKAHLPPSRLPS
CODH-L_{gbpNC}	LKAHLPPSRLPSGGGGGMNAPVQDAEARELALAGMRPRACAKEDARFIQGKGNYVDDI<br>KMPGMLHMDIVRAPIAHGRIKKIHKDAALAMPGVHAVLTAEDLKPLKLHWMPTLAGDVAA<br>VLADEKVHFQMQEVAIVIADDRYIAADAVEAVKVEYDELPVVIDPIDALKPDAPVLREDLA<br>GKTSGAHGPREHHNHIFTWGAGDKAATDAVFANAPVTVSQHMYYPRVHPCPLETCGCVAS<br>FDPIKGDLTTYITSQAPHVVRTVVSMLSGIPESKVRIVSPDIGGGFGNKVGIYPGYVCAIVASI<br>VLGRPVKWVEDRVENISTTAFARDYHMDGELAATPDGKILGLRVNVVADHGAFDACADPT<br>KFPAGLFHICSGSYDIPRAHCSVKGVYTNKAPGGVAYRCSFRVTEAVYLIERMVDVLAQKLN<br>MDKAEIRAKNFIRKEQFPYTTQFGFEYDSGDYHTALKKVLDAVDYPAWRAEQAARRADPN<br>SPTLMGIGLVTFTEVVGAGPSKMCDILGVGMFDSCEIRIHPTGSAIARMGTITQGQGHQTTY<br>AQIIATELGIPSEVIQVEEGDTSTAPYGLGTYGSRSTPVAGAAIALAARKIHAKARKIAAHML<br>EVNENDLDWEVDRFKVKGDDSKFKTMADIAWQAYHQPPAGLEPGLEAVHYYDPPNFTYPF<br>GIYLCVVDIDRATGETKVRRFYALDDCGTRINPMIIEGQIHGGLTEGYAVAMGQQMPFDAQG<br>NLLGNTLMDYFLPTAVETPHWETDHTVTPSPHHPIGAKGVAESPHVGSIPTFTAAVVDAFAH<br>VGVTHLDMPHTSYRVWKSLKEHNLALGGGGGLKAHLPPSRLPS

*Note: Gold color indicates gold-binding peptide and magenta indicates glycine.

Supplementary Figures

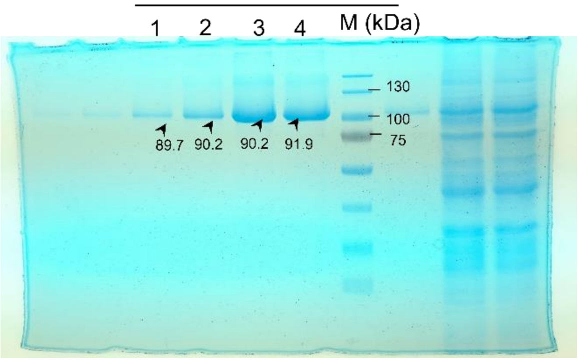

Supplementary Figure 1. Uncropped and unedited SDS-PAGE image of native and synthetic CODH-L.

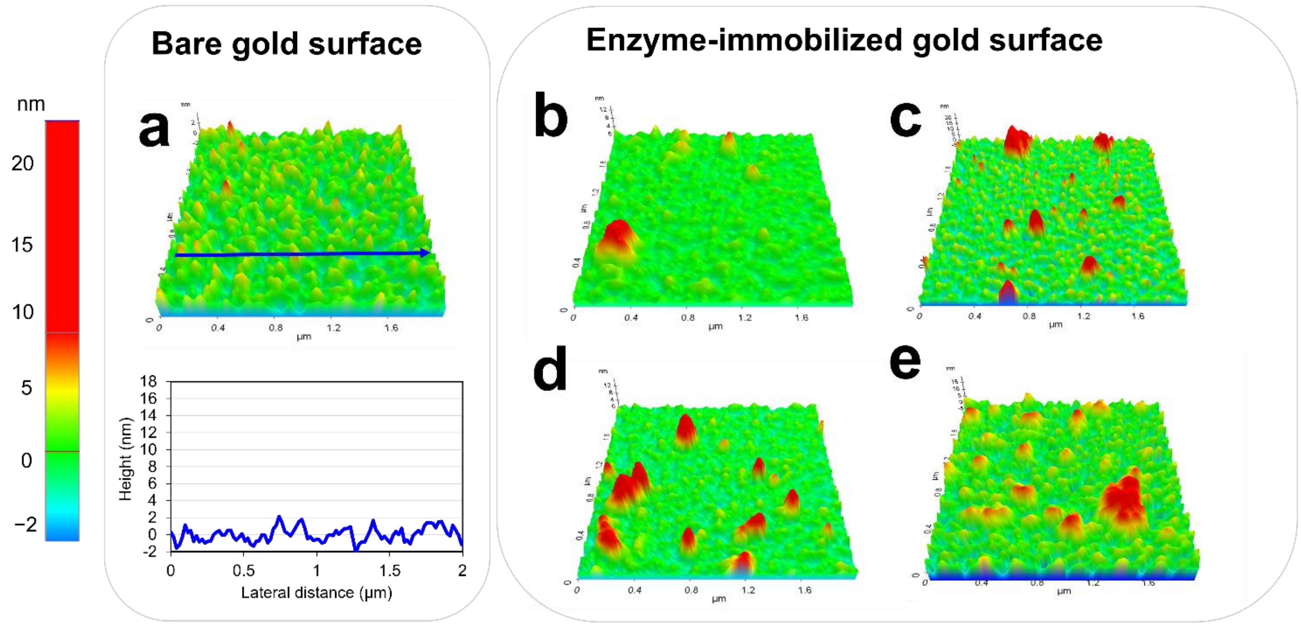

Supplementary Figure 2. Three-dimensional images of **a** bare gold surface and the cross-sectional profile along the line (lower panel), **b** native CODH-L, **c** CODH-L_{gbpN}, **d** CODH-L_{gbpN}, and **e** CODH-L_{gbpNC} when 0.5 μM of enzyme solution was incubated with gold substrate.

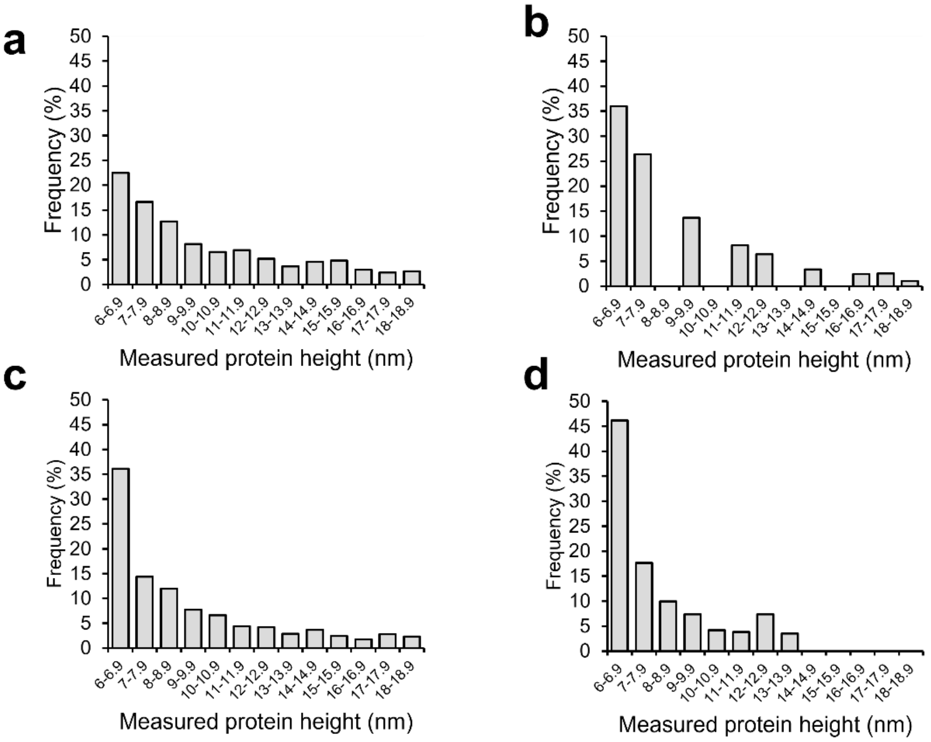

Supplementary Figure 3. Height histograms obtained from the AFM images for **a** native CODH-L, **b** CODH-L$_{gbpN}$, **c** CODH-L$_{gbpC}$, and **d** CODH-L$_{gbpNC}$ when of 1 μM of enzyme solution was incubated with gold substrate.

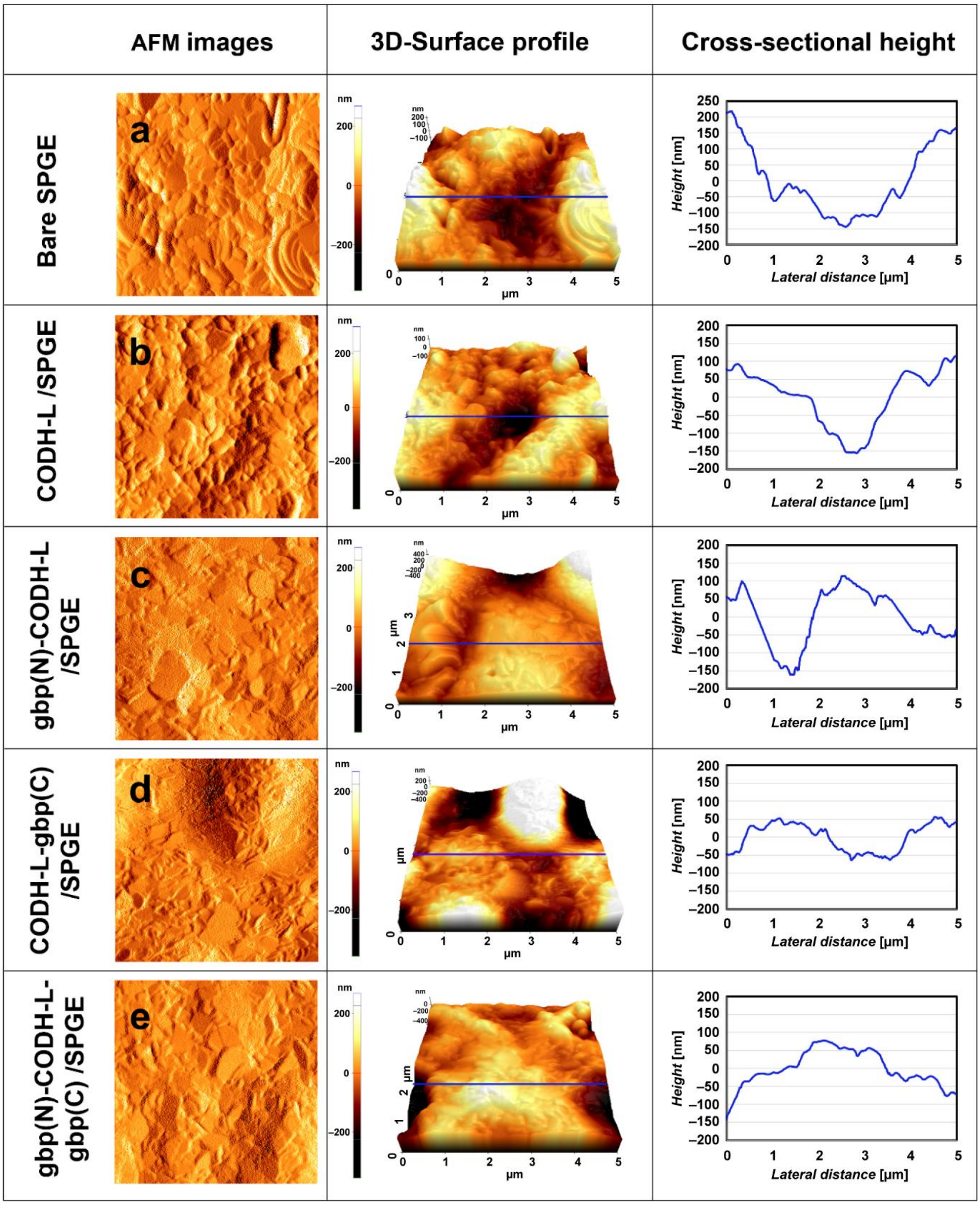

Supplementary Figure 4. AFM images, 3D-surface profiles, and cross-sectional heights of **a** the bare screen-printed gold electrode surface (SPGE), **b** native CODH-L, **c** CODH-L_{gbpN}, **d** CODH-L_{gbpC}, and **e** CODH-L_{gbpNC} on the SPGE.

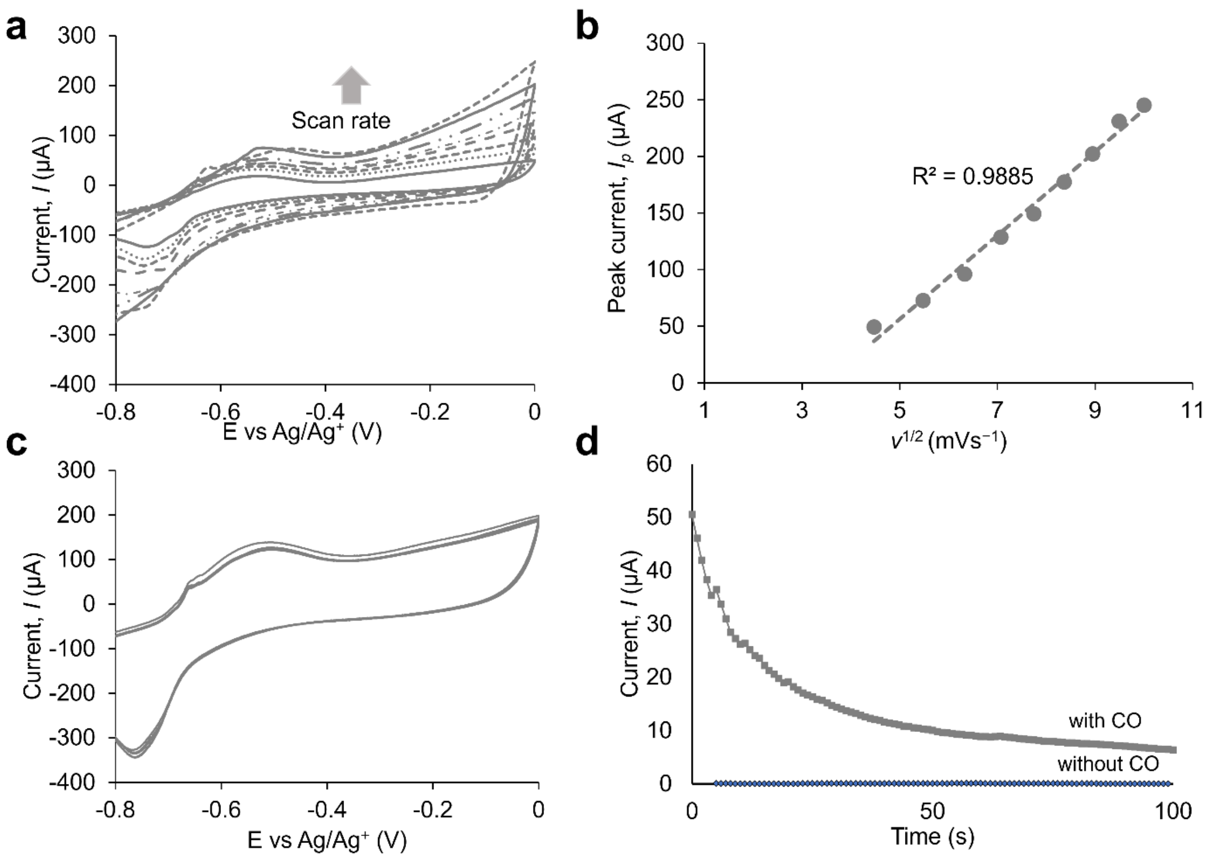

Supplementary Figure 5. a Representative CV profile of CODH-L_{gbpNC} showing the dependence of the DET current on the scan rates (scan rates: 20 to 100 mVs−1). **b** Linear dependence of peak current (at 0 V) with the square root of the scan rate. **c** CV profiles of the CODH-L_{gbpNC} when scanned continuously over 5 cycles in the presence of CO. **d** Amperometric response at an applied potential of −0.1 V (vs Ag/Ag^{+}) in the absence (blue diamond) and in the presence of CO (grey line).

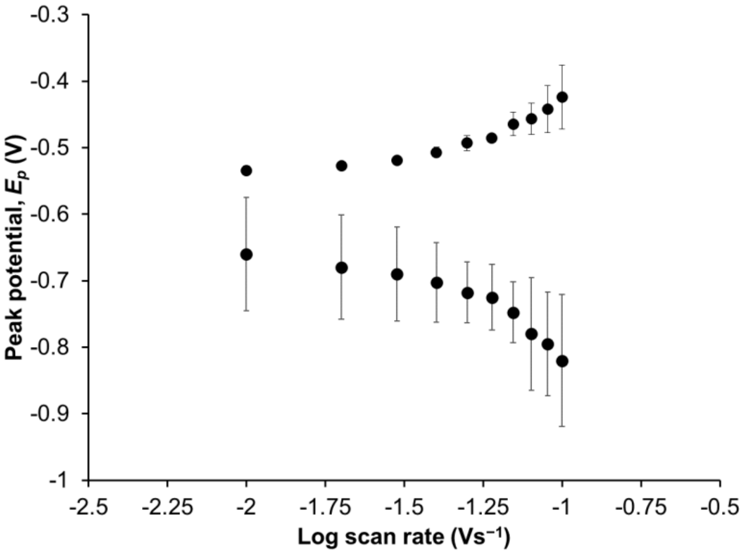

Supplementary Figure 6. Variation of peak potential, E_p against the logarithm of scan rates, log *v*.

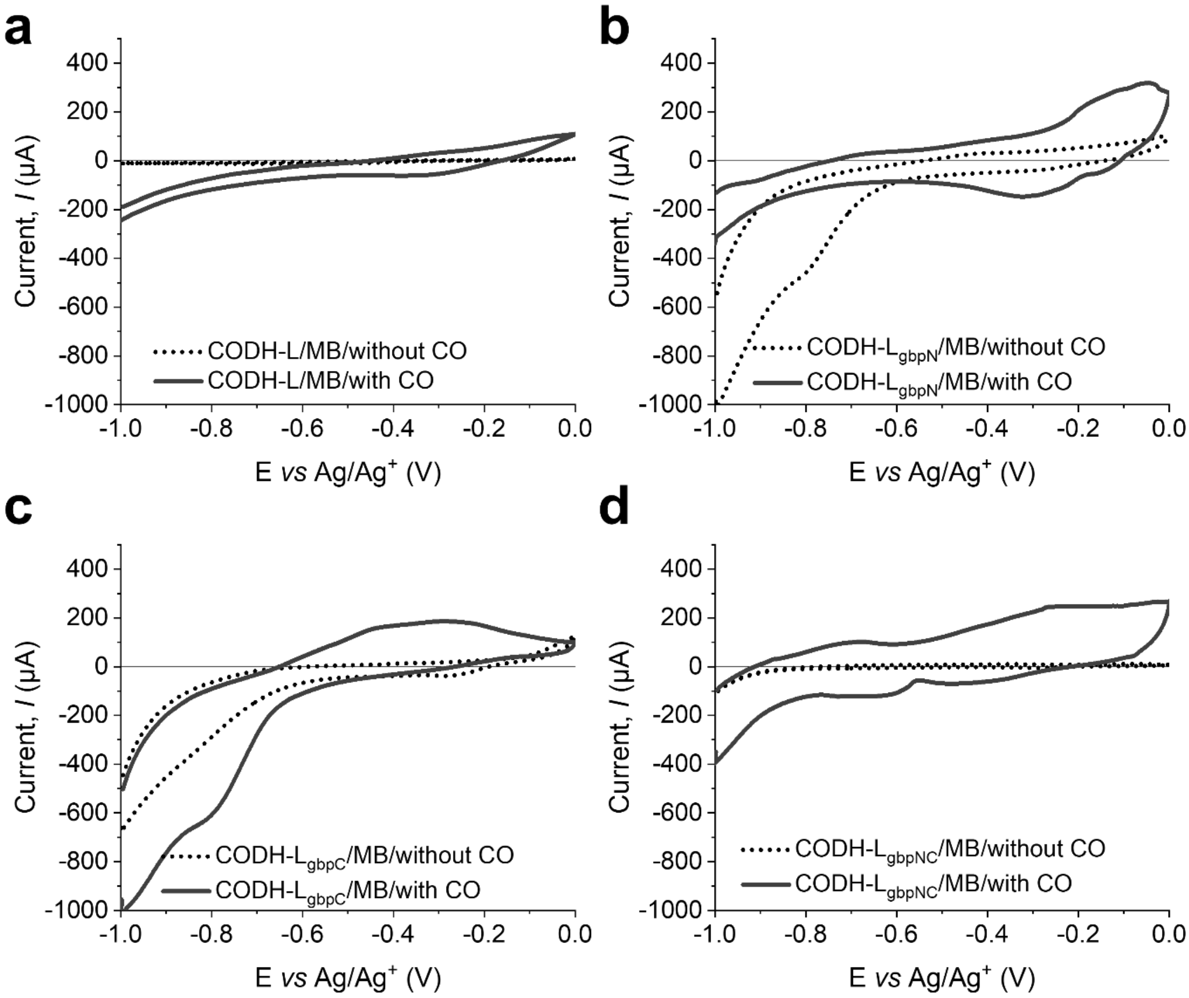

Supplementary Figure 7. CV profiles of **a** native CODH-L, **b** CODH-L$_{gbpN}$, **c** CODH-L$_{gbpC}$, and **d** CODH-L$_{gbpNC}$ on the SPGE when methylene blue was added as the redox mediator in the absence (dot line) and presence of CO (solid line).
