# Supplementary material for: Control of carbon monoxide dehydrogenase orientation by site-specific immobilization enables direct electrical contact between enzyme cofactor and solid surface
Source: Commun Biol. 2022 Apr 26;5:390. doi: 10.1038/s42003-022-03335-7 (PMC9042819; doi:10.1038/s42003-022-03335-7)
Supplement: Supplementary file 3 — Description of Additional Supplementary Files [file 42003_2022_3335_MOESM3_ESM.pdf]

Description of Additional Supplementary Files

File name: Supplementary Data
Description: Source data for underlying the graphs and plots in the main figures.

File name: Supplementary Movie
Description: Prediction of enzyme orientation on gold surface and the cofactor-surface distance.
The enzyme orientation on the electrode surface, which is regulated by the gbp fusion site, determines the distance between the cofactor and the electrode surface.
